# A fiber optic approach for cement placement and hydration assessment of deep geothermal boreholes

**DOI:** 10.1038/s41598-025-95588-5

**Published:** 2025-04-02

**Authors:** Johannes Hart, Berker Polat, Christopher Wollin, Martin Lipus, Felix Schölderle, Toni Ledig, Philippe Jousset, Thomas Reinsch, Charlotte M. Krawczyk

**Affiliations:** 1 GFZ Helmholtz Centre for Geosciences, Telegrafenberg, 14473 Potsdam, Germany; 2 Institute for Applied Geosciences, Technical University Berlin, Ernst-Reuter Platz 1, 10587 Berlin, Germany; 3 Fraunhofer IEG, Fraunhofer Research Institution for Energy Infrastructures and Geotechnologies IEG, Am Hochschulcampus 1, 44801 Bochum, Germany; 4 Chair for Hydrogeology, Technical University Munich, Arcisstr. 21, 80333 Munich, Germany; 5 Stadtwerke München GmbH, Renewable Energies, Emmy-Noether-Str. 2, 80992 Munich, Germany; 6 Institute for Geology, Mineralogy and Geophysics, Ruhr University Bochum, 44801 Bochum, Germany

**Keywords:** Fiber optic, DAS, Well integrity, Displacement efficiency, Cement hydration, Real-time monitoring, Environmental sciences, Optics and photonics

## Abstract

Achieving well integrity is mandatory for a geothermal well’s safe and sustainable operation. One of the most critical steps is the success of the primary cementing. Conventional monitoring only shows discrete snapshots after completion of the cement job. However, optical fiber sensors enable monitoring of the entire cementing process. Here, we investigate the cement placement and early hydration for a surface casing at a geothermal site in Munich, Germany. We show that distributed dynamic strain rate sensing (DDSS or DAS) allows for tracking rising fluid interfaces, determining the setting time of cement, and assessing the cement job’s success at each depth. We used DDSS and DTS (distributed temperature sensing) with a fiber optic cable permanently deployed behind the casing and combined the results with operational data, a model for the rise of fluids in the borehole, and laboratory experiments to estimate the cement setting phase. Our approach enables monitoring all phases of primary cementing, which can increase the success rate of achieving well integrity. Furthermore, it can reduce costs and improve society’s acceptance of deep geothermal wells in urban areas.

## Introduction

In the scope of green energy transition, geothermal energy production and underground thermal storage play an essential role, and many new wells need to be drilled^1^. Despite the vast experience in the petroleum industry, well integrity-related problems are a common worldwide phenomenon and result in cost-intensive work over^2,3^. While oil and gas wells are often located in remote areas, geothermal wells must be drilled near urban areas and civil infrastructure. Furthermore, different rock types, reservoir pressures, changing temperature loads, and different well designs lead to higher requirements for achieving well integrity for geothermal wells^4^. Achieving well integrity, which means no flow paths behind the casing and a casing protected from corrosion and mechanical stress, makes primary cementing one of the most critical operations within the construction of the well^5,6^.

Once the hole is drilled, the caliper is measured (with a caliper log), and the casing is deployed. The drilling mud is circulated to clean the borehole (conditioning of the drilling mud), and the cementing pipe is attached to the bottom of the casing. The following cement job consists of the cement placement and the cement setting and is controlled by the chosen cement composition’s hydration process. The exothermic process from liquid slurry to solid cement consists of five phases (I–V), reflected in the characteristic release of hydration heat^7^. Hydration of the same cement composition can be influenced by, e.g., surrounding curing temperatures, applied pressures, or the water-cement ratio^8^.

*Cement placement* Once the cement is mixed with the required water, the hydration starts a brief initial reaction (induction phase, I). The following dormant phase (II) is designed to prevent the slurry from solidifying as long as it is pumped in place. In the pumping process, the drilling mud in the annulus between the casing and the formation has to be entirely displaced by cement rising from the bottom to the surface. Contaminations of the cement can occur due to drilling mud residuals or an unstable interface between the different fluids^9^. A spacer and wash can be pumped between the drilling mud and the cement to reduce the risk of contamination and improve displacement efficiency (ratio of annulus volume and displaced volume of drilling mud). Influences on displacement efficiency include e.g., displacement technology, rheological properties, density, flow regime, pipe movement, and eccentricity^10^. The rise of the cement is controlled by monitoring the pumping parameters (rate, pressure, volume, and density). Further, the drill rig measures the pressure and return flow rate. These parameters show an average value for the conditions along the entire borehole and can be compared with commercial borehole simulators, which might include a model for fluid displacement^11,12^.

*Cement setting* The phase change of the cement occurs in the acceleration phase (III) once the cement slurry is in place. In this phase, the cement slurry reaches the so-called initial set (beginning of solidification) and final set (complete solidification)^13–15^. The hydration enters the deceleration phase (IV) with decreasing reaction kinetics and decreasing released heat. While hardening continues at a very low level in the steady phase (V), we focus this work on the 24 h of early hydration. The cement must withstand restarting drilling operations to construct the well’s next section. The time until this strength is called wait on cement (WOC). An ultrasonic cement analyzer test (UCA Test) can be performed beforehand to estimate the minimum WOC. This acoustic test determines the compressive strength evolution with estimated downhole temperature conditions. Experiences showed that this temperature estimation is often inaccurate, resulting in an inaccurate prediction of the WOC^16^. In addition, a reference sample of the pumped cement slurry is observed at the surface.

Once the cement is in place and set, the success has to be proven by geophysical downhole logging, hydraulic pressure tests, and a volumetric balance of the annulus and the pumped cement^5^. Downhole logging usually includes temperature measurements that indicate the desired height of the top of cement (TOC) and acoustic measurements, e.g., Cement bond logs and UltraSonic imager tools. These acoustic methods use sonic and ultrasonic waves to determine quantitative or even segmented information about the quality of the cement. It should be noted that these measurements can only be taken after cementing. Further, the results of these acoustic methods depend heavily on various input parameters and can be ambiguous^17,18^. Despite the experience of millions of drilled deep boreholes and continuous technological improvements to reach well integrity, leakage of wells is still a widely observed phenomenon, with an urgent need for further improvement. An investigation of almost 1000 conventional and unconventional onshore wells in Canada showed, e.g., that 28–32% were not pressure tight^2^. Next to environmental concerns, an integer well is also economically of great interest. Higher reservoir pressures, fewer shut-ins, and less expensive workovers increase productivity significantly^19^.

The DDSS technology demonstrated its added value in various geoscience-related topics, including urban exploration^20–22^, natural hazards^23^, and reservoir characterization^24–27^. This technology also proved its ability to track ultra-slow phase changes, providing information on absolute strain and temperature changes^28–30^.

Within boreholes, several successful fiber optic campaigns evaluated well integrity-related processes, such as cement placement^31–33^, cement hydration^16,31,32^, quality of the cement behind casing^31,34^, the integrity of the casing^35^ and the integrity for a well after plug and abandonment^36^. However, an approach to monitor displacement efficiency and the end of the cement setting time with DDSS for each depth is still missing.

Here, we show that fiber sensors allow the primary cementing process to be monitored. We develop an approach to continuously track the rise of different fluid interfaces and evaluate the displacement efficiency for each depth. In this approach, we developed a fluid displacement model to compare the observed fiber sensor data. We further demonstrate that DDSS measurements allow us to determine the end of the cement setting time and, consequently, the WOC. With this information, we can assess each depth’s cement quality behind the casing.

To demonstrate this, we monitored the primary cementing of an 874 m surface casing at a geothermal site in Munich, Germany (Methods: Experimental Setup). We measured DDSS and DTS with a fiber optic cable permanently deployed behind the casing. We compared the observations with a fluid displacement model and the estimated WOC based on laboratory experiments. By controlling the displacement efficiency in real-time and knowing the hydration process, we see the potential for a significant increase in achieving well integrity. This can increase society’s acceptance of deep boreholes in urban areas.

## Results

### Initial borehole temperature conditions

**Fig. 1:**
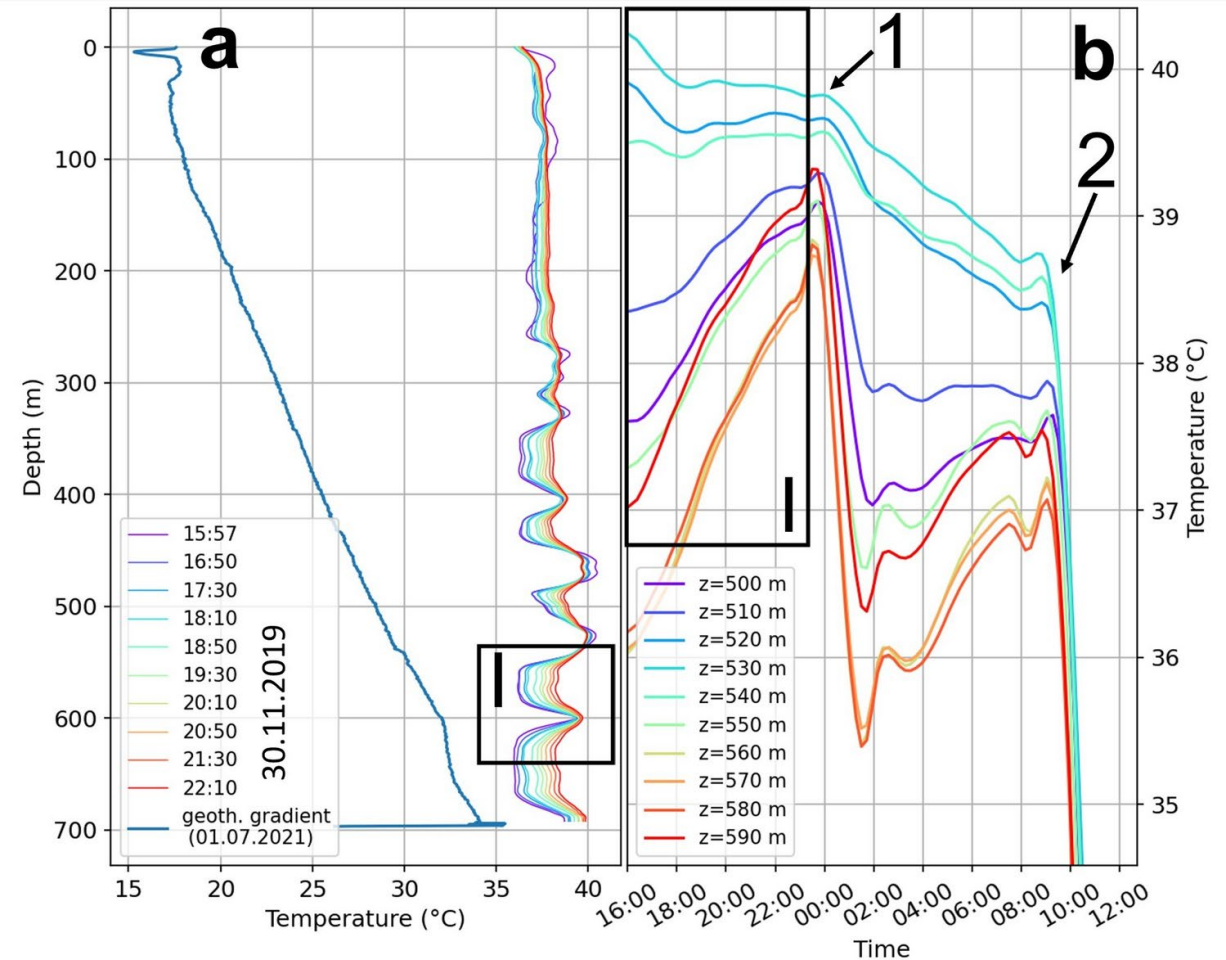
Initial temperature conditions within the borehole. (**a**) Temperature in the borehole as a function of depth on the 30.11.2019 between 15:57 and 22:1 in comparison with the natural temperature (known from the geothermal gradient measured on the 01.07.2021). The temperature mostly rises along the whole depth, despite the significantly lower natural temperature, indicating a drilling induced strongly heated up borehole. Some depths (e.g., 520–540 m) slightly cool down (highlighted in I). (**b**) Temperature evolution with time for depths between 500 and 590 m. Depths of 520–540 m cool down less when drilling mud is circulated within the casing (1). Once the circulation goes through the annulus between casing and formation all depths cool down (2). These anomalies show most likely section where the fiber sensor is not firmly attached to casing.

**Fig. 2:**
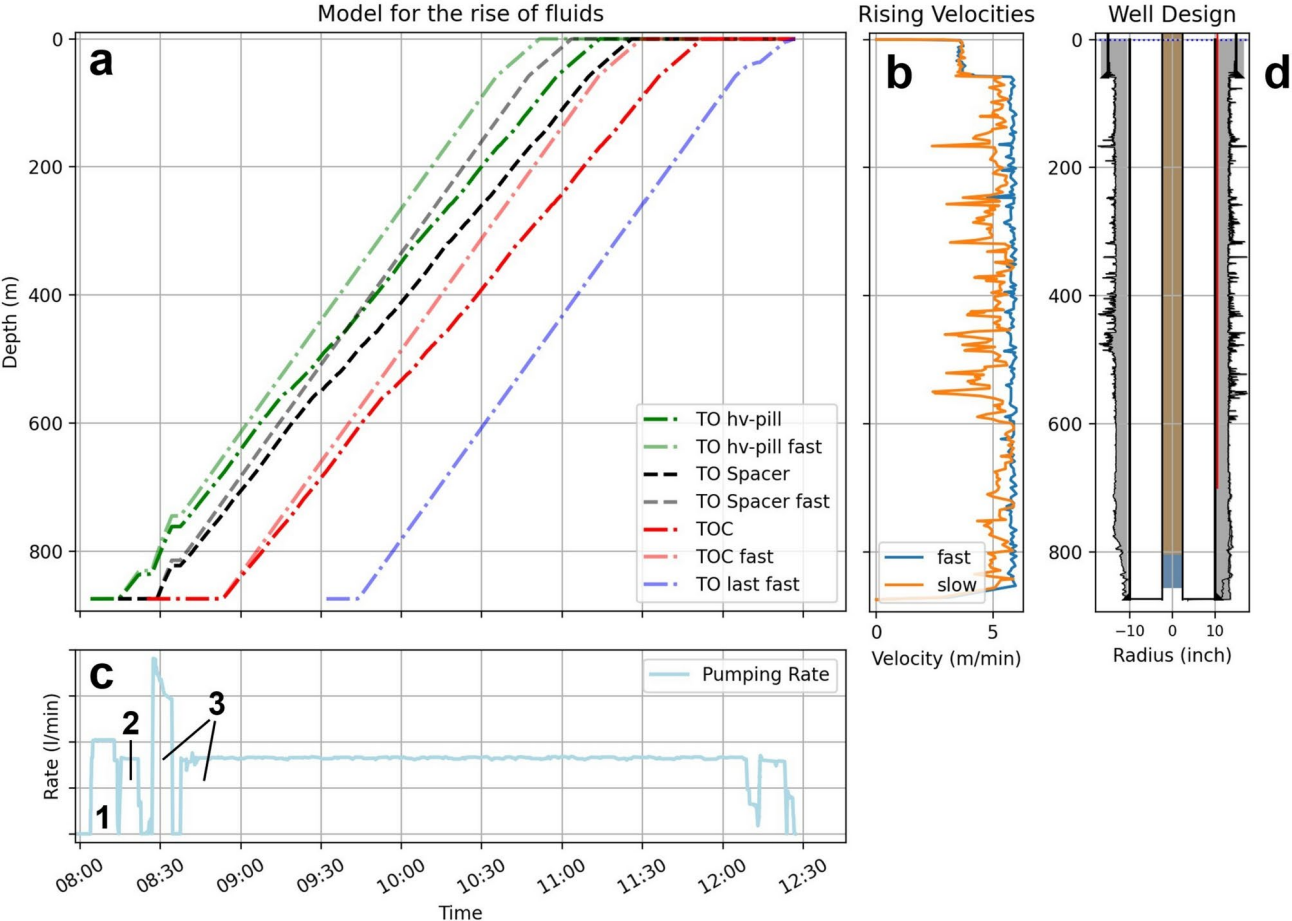
Results of the fluid displacement model. (**a**) Temporal evolution of the different fluid interfaces (HV-pill: TO hv-pill and TO hv-pill fast, freshwater spacer: TO spacer and TO spacer fast, the rise of the cement: TOC and TOC fast and the last rise linked to the end of the pumping process: TO last fast). Depending on the borehole geometry, the arrival of the different fluids at the earth’s surface differs by around 30 min. (**b**) Predicted rising velocities with depth. While the rising velocity with the ideal borehole geometry is almost constant at 6 m/min, it fluctuates between 2.5 and 6 m/min due to the borehole breakouts. (**c**) The field data of the pumping service shows pumping of the high viscosity pill (1), the freshwater spacer (2) and the cement (3). (**d**) The well design.

**Fig. 3:**
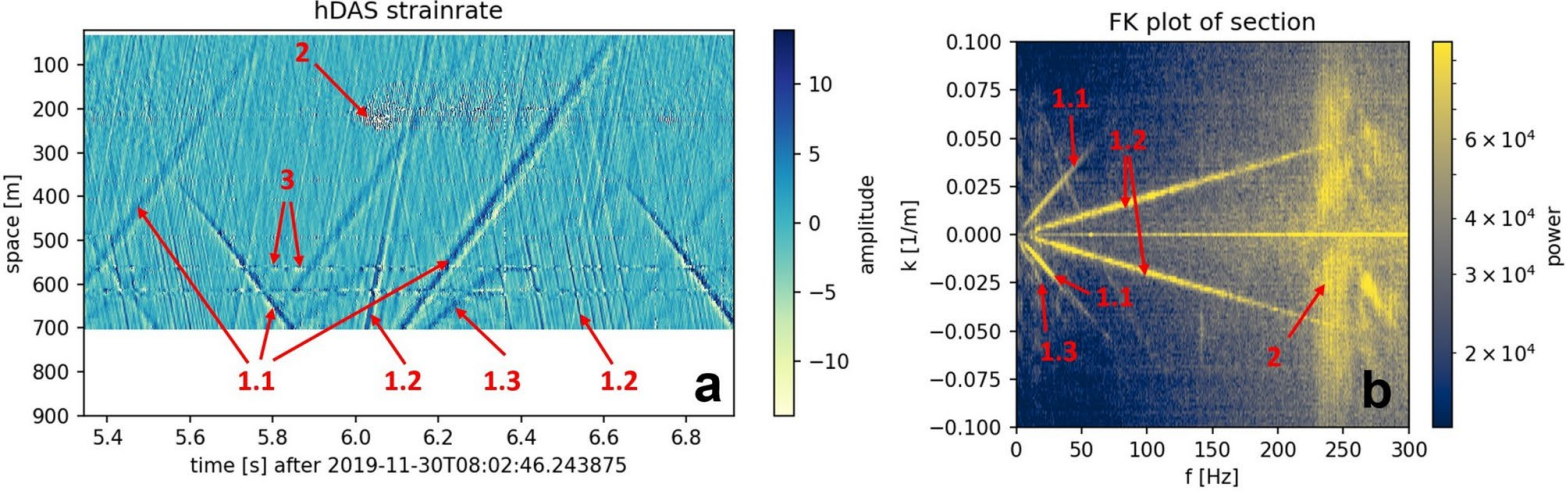
1.5-s snapshots of the strain rate data while cement placement. (**a**) Distributed strain rate as a function of time and depth and (**b**) as a function of frequency and wavenumber. Three wave modes are propagating along the borehole (arrow 1.1–1,3). Further, local and temporal impacts with small move-out distances (arrow 2) and reverberations with no move-out (arrow 3) are evident. Mode 1.1 (5–90 Hz, v = 1250–1350 m/s) has characteristics of tube waves. Mode 1.2 (20–300 Hz, v = 4900–5200 m/s) are most likely guided by the casing. Waves of mode 1.3 are only observed upgoing with a velocity decrease from 300 to 500 m/s.

To understand the influence of the formation temperature on the cementing process, we evaluated the initial temperature conditions before the start of the cement job. We compared them to the geothermal gradient^37^ to understand the formation’s influence on the cementing operation (Fig. 1a). Most parts of the borehole warm up by up to 2.5°K despite the significantly lower temperature of the natural, undisturbed temperature known from the geothermal gradient. We consider a drilling-induced heated-up near-field of the wellbore as a likely reason. The field reports show that the mud’s outflow reached temperatures of up to 67 °C during drilling in this section. The warming indicates a pumping operation with a cooling effect before our measurement started. In several locations, e.g., 530 m, cooling up to 1°K occurs instead (Fig. 1a, b, box I). As indicated by arrow 1, rapid cooling of around 5°K coincides with the pumping process to clean the drilling mud (Fig. 1b), which again does not influence depths like 530 m. Only when the fluids start circulating through the annulus at the beginning of the pumping operation do all traces cool down (Fig. 1b, 2). In these intervals, the cable is probably not firmly attached to the casing, resulting in a lack of coupling between the cooling inside the casing and the cable. At these depths, continuous noise occurs during pumping in the strain rate data (Fig. 3a, 3), indicating that the cable has less tension in these depth intervals.

### Estimating the rise of fluid interfaces

We developed an analytical model to predict the rise of stable fluid interfaces in the annulus between casing and formation (Methods: Fluid displacement model). We tested the rise for the top of various fluids (Fig. 2). We calculated the fluid rise for the minimum volume of the annulus (drill bit diameter with no breakouts) and the maximum volume (accounting for all breakouts from the caliper log).

The model results in the expected rise of each fluid interface (Fig. 2a) and the rising velocity for each depth (Fig. 2b) based on the cementing service’s pumping rate (Fig. 2c). With the minimum annulus (the ideal drill bit diameter), the fluid rise in the annulus depends only on the pumping rate and has a velocity of around 5.9 m/min and reaches the surface within 2:35 h (further called “fast rise”). Considering the borehole geometry based on the caliper log, the rising velocity ranges between 2.4 and 5.9 m/min, resulting in an ascent to the surface after 2:59 h. Deviations from the expected cement rise can show depth-dependent anomalies, which may jeopardize the cement job’s success and the well’s future integrity. A faster rise than expected indicates that the annular void space has not been filled, which might occur, e.g., due to gelled mud within the washouts^10^. A slower rise than expected would provide indications of loss zones or new borehole breakouts^10^. Consequently, an accurate comparison might even allow quantifying the amount of, e.g., undisplaced mud/residual filter cake.

### Wait on cement estimation

We used the results of laboratory experiments to understand the hydration of the chosen cement composition. The cementing services UCA-Test shows the evolution of the compressive strength and provides an estimated minimum Wait on Cement. We further conducted a shear-wave (S-wave) velocity experiment (Methods: Wait on Cement Estimation). With the onset and increase of the S-wave velocity, we estimate the beginning and evolution of solidification.

The UCA-Test showed that the cement reaches the mandatory compressive strength (> 500 psi^38^) after roughly 8 h. The evolution of shear wave velocity reveals an onset 8–12 h after mixing. Based on this experimental data, we define a minimum WOC of 12 h. However, as the cement is mixed continuously along the whole cement job, the mixing time for the cement differs for each depth. We use the fluid displacement model fast rise results to understand the duration of one pumping circulation cycle. We conclude that the cement close to the surface was mixed at the beginning and the cement on the bottom at the end of the last cycle (Methods: Wait on Cement Estimation).

### Cement placement

We demonstrate downhole tracking of rising fluid interfaces with DDSS by comparing low-frequency responses in the dynamic strain rate with the modeled rise of fluids in the annulus. The drilling mud has to be entirely displaced by cement to avoid any flow paths behind the casing. Usually, this process is solely controlled by comparing the predicted rise of fluids with the pumping parameters of the cementing service^10^.

#### Distributed temperature sensing

DTS technology allows us to find temperature-related indications for the movement of fluids colder than the formation temperature. A slight cool-down, followed by a warm-up and temperature drop of 15°K at the end of the sensing cable, indicates the arrival of the cold cement slurry (Fig. 5a). This cold front moves continuously upwards with decreasing intensity (at 100 m, the temperature drops 3°K). However, the sensed rise of the cold front differs from any modeled fluid rise. One explanation for the offset could be the fiber’s thermal response. However, little work has been published about the thermal response time of solid and rigid downhole cables (e.g., our flat pack cable, 13.7 × 30.5 mm). Solving the one-dimensional transient heat conduction problem^39^ indicates a response time on a minute scale for 95% of the temperature change. Detecting the effect becomes more challenging for minor temperature differences, and an in-depth investigation with an appropriate modeling framework is recommended. The cable might be firmly attached to the casing in depths, which showed less sensitivity when cooling (Fig. 5a). The 20" casing is filled with drilling mud, which needs time to cool down and could counter the cool down in these specific locations.

#### Distributed dynamic strain sensing

Most acoustic energy is coupled to the borehole by the pumping process and works on the rig floor. A representative snapshot of the strain rate data with its corresponding FK plot provides a full picture of the partly not understood subsurface wavefield (Fig. 3). It shows guided waves in three modes traveling likely along the borehole axis (Fig. 3, arrow 1.1–1.3). Their occurrence, amplitude, and origin vary during cement placement. Waves of mode 1.1 (5–90 Hz, v = 1250–1350 m/s) have typical characteristics for so-called tube waves^40^. These waves travel in the fluid column within the casing, as seen, e.g., in fiber optic vertical seismic profiling^41^. Waves of mode 1.2 (20–300 Hz, v = 4900–5200 m/s) are most likely guided by steel and confined to the cross-section of the casing or the stinger^42^. The mode 1.3 waves (5–20 Hz, v = 500–300 m/s) appear only upgoing with a decreasing speed. These waves are generated at the bottom of the borehole upon the arrival of tube waves and could result from a tube wave to shear wave conversion. This mode’s 5–20 Hz frequency range backs this assumption, as it is almost a magnitude smaller than the range of the tube waves, a typical feature for this conversion^43^. Since this feature shows a decreasing speed with distance and does not co-occur on all channels, we do not assume it is an artifact due to the measurement principle. In addition to propagating waves, local and temporal impacts with small move-outs, usually less than 100 m (Fig. 3, arrow 2), and depth-stable noise with no visible move-outs (Fig. 3a, arrow 3) occur.

**Fig. 4:**
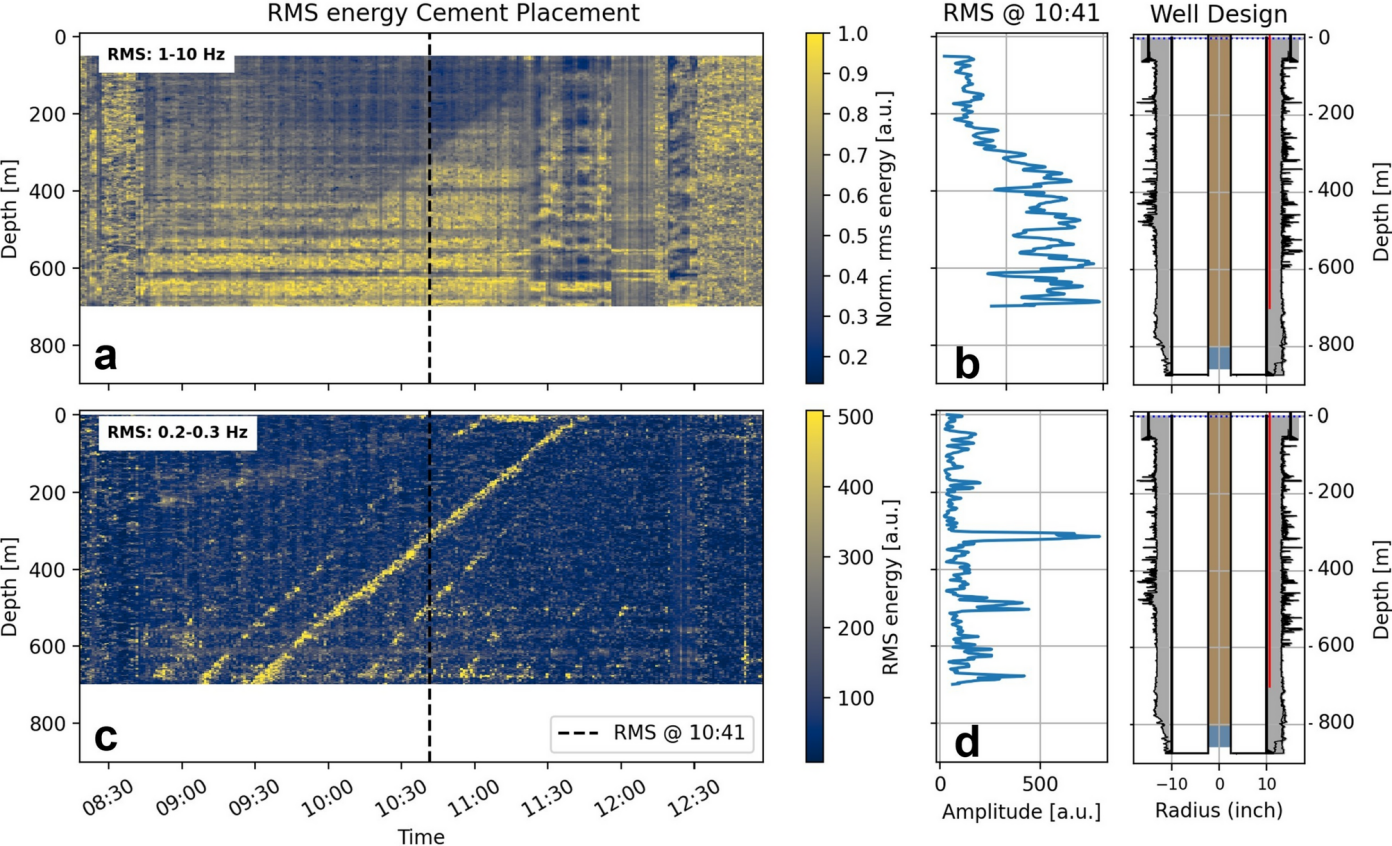
Vibrational energy from dynamic strain rate while cement placement. (**a**, **b**) shows the vibrational energy level in 1–10 Hz, as a function of time and depth (**a**) and as a function of depth at 10:41. The vibrational energy level rises once the cement reaches the sensing cable. (**c**, **d**) shows the vibrational energy in a frequency range of 0.2–0.3 Hz as a function of time and depth (**c**) and as a function of depth at 10:41(**d**). Line-like features with different slopes appear in the 0.2–0.3 Hz. These features are peaks in the vibrational energy.

We summarized the strain-rate data for different frequency bands with a root mean square approach to obtain the vibrational energy (Fig. 4, Methods: DDSS). The vibrational energy in a frequency range of 1–10 Hz (Fig. 4a) shows between 10:00 and 11:15 a transition between a lower (shallow) and higher energy level (deep), as seen in detail in the example at 10:41 (Fig. 4b). We cannot assign any mode of waves or impacts to the rising features. The elevated vibrational energy seems to be hidden in the noise and might be a response to the particles in the slurry, resulting in higher friction on the cable. The extent of the transition zone to the elevated energy level (Fig. 4b) may even provide information about the extent of the mixing zone between the spacer and cement.

Lower frequency ranges revealed several line-like low-frequency features with different slopes, particularly pronounced in 0.2–0.3 Hz (Fig. 4c). These features show a transient peak in the vibrational energy (Fig. 4d). We assume these features visualize a fluid’s physical property change or a low-frequency response to temperature or pressure changes^29,30^.

#### Rise of fluids

**Fig. 5:**
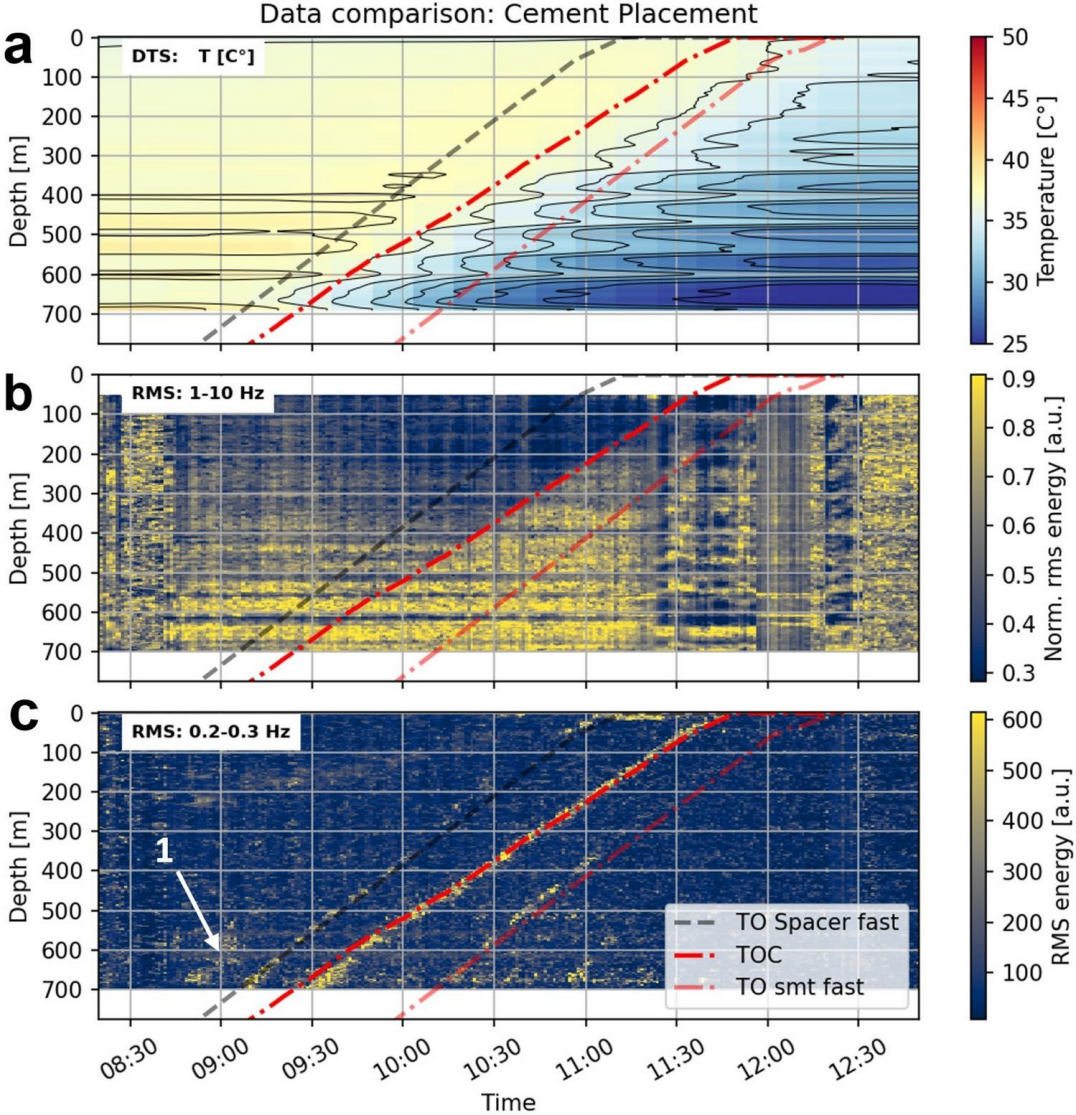
Combined interpretation of the Cement Placement. (**a**) The temperature evolution as function of time and depth. DTS shows that the cold front rises slower than any modeled rise. As heat transfer is a comparably slow process, this technology does not allow for tracking the rise of cement accurately. (**b**) The vibrational energy in a frequency range of 1–10 Hz as a function of time and depths shows the transition to the elevated energy level and matches the estimated slow rise of the cement head (Top Of Cement, TOC). (**c**) The vibrational energy in a frequency range of 0.2–0.3Hz as a function of time and depths shows that the predicted rise matches the most dominant low-frequency. Further, the slope of the first low-frequency response matches the fast rise of the spacer (TO Spacer fast, fast for fast rise). The subsequent line-like features are slightly steeper than the predicted fast rise (TO smt fast).

We combine the results of our fluid displacement model with the measured fiber optic data (Fig. 5). The slope of the cold front in the temperature data is lower than any modeled rises, indicating that relying solely on absolute temperature readings can lead to inaccuracies in identifying the interface movement (Fig. 5a). While the thermal response could explain the offset on the one hand, the heat transfer between formation and cement could further explain the deviation of the slope. The cement is in contact with the initially hotter formation fluid, which, over time, cools down. This could reduce the thermal fingerprint of the top of the cement, hence misinterpreting the actual depth. Although the thermal data potentially contains all the necessary information to characterize the cold front, it was not fully utilized in our current analysis. In principle, the fraction of the expected thermal step at a given depth could be harnessed, either in post-processing or in real-time, provided that a suitable modeling framework is in place. However, as demonstrated in the following chapter, extracting these insights from temperature data is more challenging than leveraging DDSS measurements.

As the temperature measurement did not reveal further details with our experiment setup (comparably low temporal sampling resolution of 13 min), we focus on the features in the vibrational energy obtained from the dynamic strain-rate measurement.

Around 8:45, scattered vibrational energy corresponds to the beginning of the expected rise of the HV-pill (Fig. 5c, arrow 1). It could be that the higher density of this pill compared to the drilling mud led to the rat hole (unknown borehole volume below the casing) being filled first. Due to the lower density of the following freshwater spacer, the pill can not be displaced. From 9:30 we see a relationship between the first traceable low-frequency response and the predicted freshwater spacer’s fast rise (Fig. 5c, TO Spacer fast). The time fits, assuming the spacer does not fill the rat hole. The predicted slope of the cement slow rise (Fig. 5, TOC) is associated with the transition to the elevated vibrational energy level (Fig. 5b) and the most dominant line-like feature (Fig. 5c). If we assume a rat hole volume of 10 m^3^, which has to be filled first, the predicted rise matches also in time. Finally, subsequent low-frequency features within the continuous cementing process revert to a fast rise and show a slope slightly steeper than the slope of the freshwater pill (Fig. 5c, TO smt fast).

#### Implication for displacement process and efficiency

**Fig. 6:**
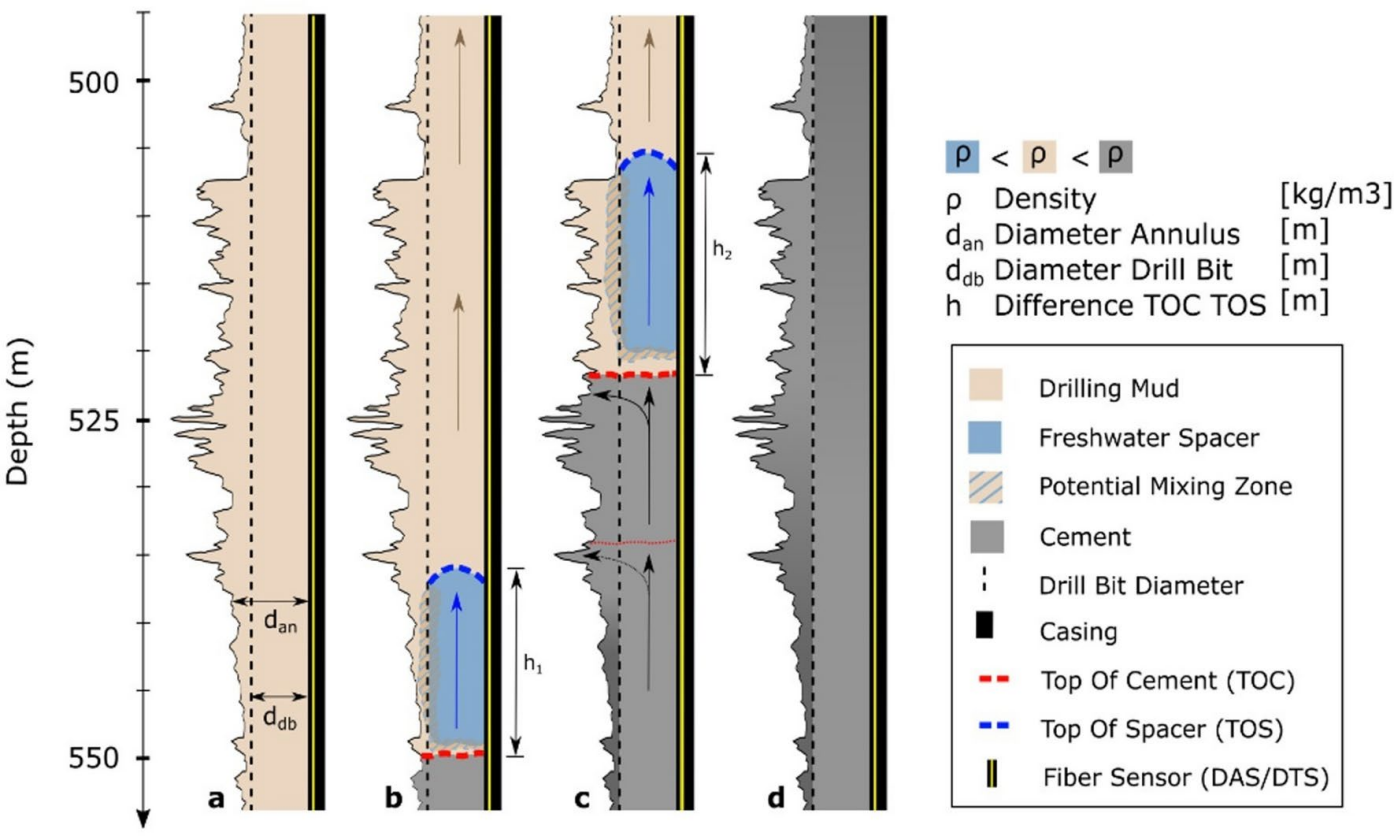
Schematic principle of fluid displacement based on two rising velocities at constant pumping rate. The figure shows each a half vertical cross-section of the borehole. (**a**) The annulus between the casing and the Formation is filled with drilling mud. (**b**) Once the cement job starts, we first sense the fast rise of the freshwater spacer. As the spacar seeks the path of least resistance it does not displace the drilling mud from the washout. Some intermixing is likely to happen. (**c**) The Top of Cement follows the spacer with an increasing distance and displaces the drilling mud due to its higher density. The succeeding cement passes the cement in the breakouts before it fills the following breakout. (**d**) Within the breakouts, the cement has an earlier mixing time the deeper it is in the borehole. Within the path of least resistance, the cement has a later mixing time the deeper it is placed in the borehole.

Our findings provide insights into the subsurface displacement process of different fluids. Figure 6 illustrates the displacement process, showing the observed differing rising velocities. Fluids with a lower density than the fluid to be displaced, e.g., the freshwater spacer compared to the drilling mud, seek the path of least resistance. We find that the smallest diameter of the annulus defines the path of least resistance for the freshwater spacer (Fig. 6a, initially the drill bit diameter d_db_). On the other hand, fluids with a higher density, such as cement, can fill the entire annulus volume between the casing and the formation (Fig. 6a, annulus diameter d_an_).

The Top of the freshwater spacer rises fast and only displaces drilling mud within the path of least resistance (Fig. 6bc). Intermixing between drilling mud and freshwater spacers most likely occurs in the washouts, but this is not reflected in the fluids volume balance. An even slightly faster slope indicates that the minimum cross-sectional area of the annulus was reduced, possibly due to residuals from drilling mud (< d_db_). The arriving cement has a higher density, uses the maximum cross-sectional area, and pushes out the drilling mud from the breakouts in front of the interface (Fig. 6b, c), which increases the distance between the mud-spacer and the spacer-cement interface (Fig. 6, h_1 _< h_2_).

As the subsequent cement has no difference in density, it again seeks the path of least resistance and passes by the filled borehole breakouts (Fig. 6c). It means that the cement inside the breakouts originates from the first cement, which has an earlier mixing time and a higher probability of contamination due to its proximity to the mixing zone. The hypothesis that the deeper the cement is in the borehole, the later it is mixed would be only valid for the cement’s flow path of least resistance. Within the breakouts, the opposite should be the case.

### Cement setting

We demonstrate the depth-accurate determination of the critical hydration phases by fiber sensors to ensure the needed WOC. We demonstrate that DDSS can solely determine the WOC. However, DTS enables a more precise understanding of the critical phases of hydration and can help to identify the causes of future well integrity problems.

#### Distributed temperature sensing

**Fig. 7:**
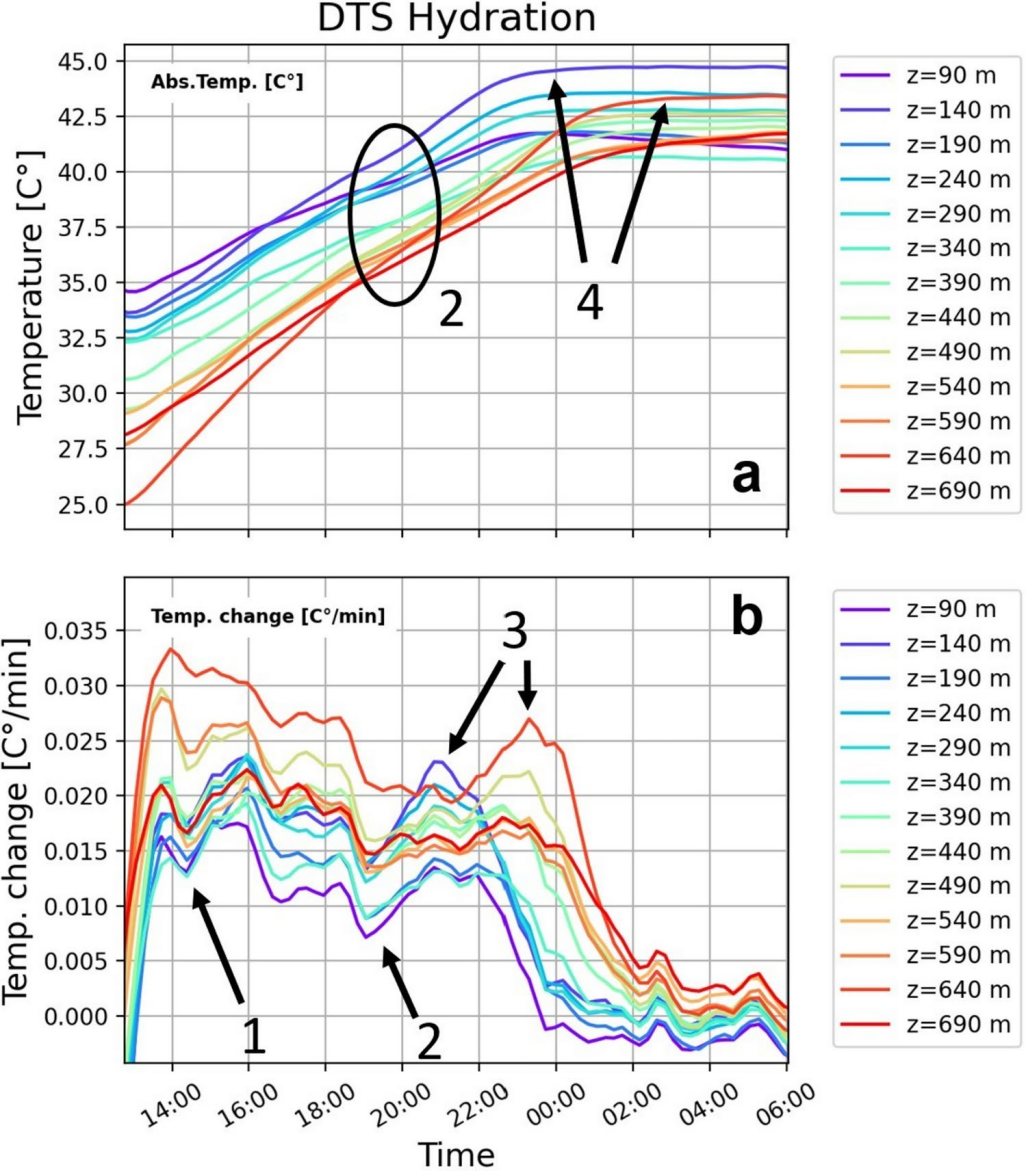
Temperature evolution of the cement in the hydration phase. (**a**) The absolute temperature evolution per minute as a function of time for depths between 90 and 690 m. The maximum temperature is reached 10 (shallow) to 14 h (deep) after the pumping process stops. (**b**) The temperature change per minute (extracted from 13 min sampling rate) as a function of time for depths between 90 and 690 m. The first maximum of the temperature change is reached for all depths at the same time (1). Each depth develops a different trend once the temperature surpasses the initial conditions (2, a). The deceleration phase of hydration begins with the drop of temperature change (3, 4). The steady phase starts once the temperature change fluctuates at very low levels.

Once the displacement stops, the entire borehole starts to heat up (Fig. 7a). The first maximum of the heat rate development is reached independently from the different mixing start, which indicates that the formation’s high temperature has initially been the primary influence on temperature evolution. (Fig. 7b, arrow 1). A concurrent decrease over the entire depth suggests that the formation’s warming effect is simultaneously decreasing due to the equalization of the temperature between the annulus and the formation. The released hydration heat dominates once temperature evolution develops differently at each depth. This conclusion is supported by the fact that this only occurs once the absolute temperature surpasses the initial borehole temperatures (Fig. 7, arrow 2). Cement at a depth of 90 m shows its last maximum temperature change up to 2 h earlier than the cement at the end of the sensing cable (Fig. 7, arrow 3).

#### Distributed dynamic strain sensing

**Fig. 8:**
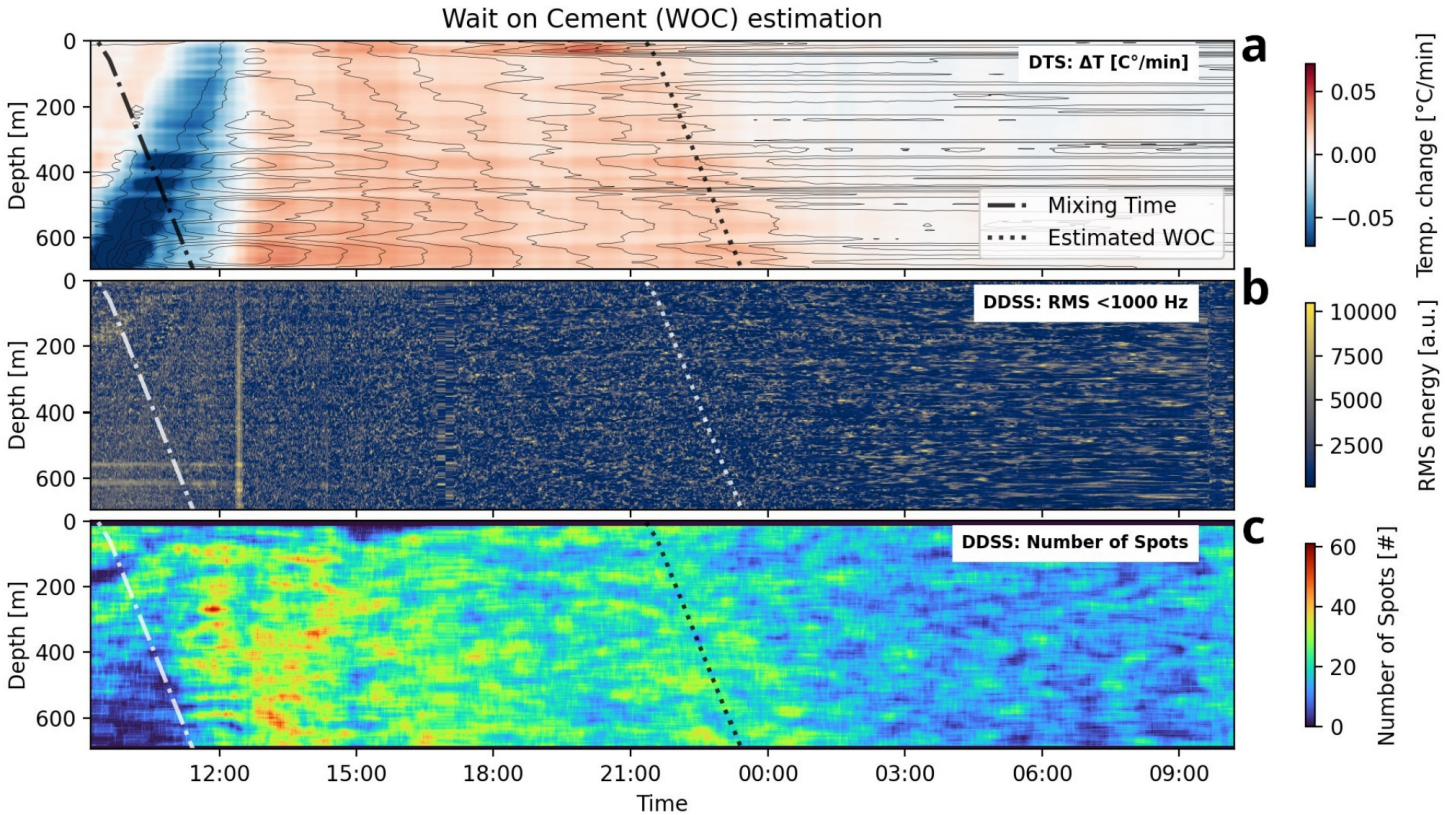
Combined observation for the cement setting. The figure shows the fiber optic data in comparison with the estimated mixing time and the estimated WOC (**a**) The temperature change as a function of time and depth. The temperature change drops with the estimated minimum WOC of 12 h. Depths where heat is generated longer correlate with borehole breakouts. (**b**) The vibrational energy in 0.2–0.3 Hz as a function of time and depth. A blotchy pattern with coarsing spots is evident. (**c**) The number of spots in a window of 30 min by 30 m around each value as a function of time and depth. The decrease in the number of spots matches with the decrease of the temperature change.

Our dynamic strain rate data shows no modes of propagating waves within the cement setting phase. Only localized events on a single channel with no move-out occur. These events appear irregularly over the depth interval and cover the entire frequency range between 0 and 500 Hz. In the vibrational energy, these events are evident as an irregular pattern of blotchy spots, which become coarser over time (Fig. 8b). Figure 8 shows a relationship between the blotchy pattern and the decrease in temperature, which suggests a phenomenon controlled by temperature changes. These features originate probably from a very local load compensation due to shrinkage or thermal cracking of the cement or thermal expansion of the casing. Further understanding of the origin of the blotchy pattern in vibrational energy is still under investigation.

#### Stage of hydration/wait on cement (WOC)

We combine the fiber optic data with our estimate for the mixing time for each depth (Methods: wait on cement estimation) and the corresponding 12 h to reach the mandatory compressive strength (Fig. 8). The transition from the acceleration phase to the deceleration phase is shown for each depth by the different start times for the last drop in the temperature change^7^. The steady phase should have begun as soon as the temperature change fluctuates at a low level. In this phase, the temperature measurement fluctuates by ± 0.05°K simultaneously over the whole depth, which may indicate the noise level of the used DTS measurement system. This low resolution could be due to the relatively short measuring length and long integration time (13 min).

The significant drop in the temperature change (between 22:00 and 01:00) matches in the upper half of the borehole to our predicted maximum waiting time of 12 h (Fig. 8a). In some depth intervals of the lower half, relevant heat is released 1–2 h past our estimated minimum WOC. These depths are usually linked to larger washouts. With a change in the released heat of hydration, the blotchy pattern of the vibrational energy changes (Fig. 8b). We quantified the appearance of the spots by determining the number of spots in a window of 30 min by 30 m around each value (Methods: DDSS). We conclude that the faster the system warms up, the more and smaller spots appear. As soon as the heating process decreases, their number decreases.

## Discussion

During the drilling and completion of deep boreholes, achieving well integrity ensures a geothermal power plant’s safe and sustainable operation. The German Association of gas, oil, and geoenergy (BVEG) defines well integrity as being achieved “when all fluids contained in a well are safely controlled at every possible combination of pressure and temperature to which they can be subjected within the intended operating conditions”^5^. The success of a cement job is proved once the cement is in place and set, which results in an information gap throughout the cement job.

This study demonstrates that fiber sensors can close information gaps in conventional downhole measurements to improve displacement efficiency, determine the critical hydration phases, and evaluate cement jobs’ success.

The longer the pumping operation lasts, the longer the temperatures in the borehole equilibrate, and the suitability for tracking the rising fluid interfaces decreases. As heat transfer is a comparably slow process, we conclude that DDSS is better suited to monitor the rise of fluid interfaces than DTS.

We can decipher most of the features in the DDSS raw data. In particular, feature 1.3 (Fig. 3, arrow 1.3) is not understood. The decrease in velocity with its ascent indicates a dependency on formation properties. These waves could be waves traveling along the interface between the fluid and the borehole wall, emitting a p-wave to the fluid in the annulus, which is then detected by the sensing cable. However, 300–500 m/s is too slow for S-waves in formations at more than 500 m depths in the Bavarian Molasse Basin^44^. Therefore, the velocity could be smaller than expected by a drilling-induced loosening of the formation in the near-field boreholes.

In contrast, the vibrational energy of the strain rate measurement allowed different fluid interfaces to be associated with low-frequency responses. Different slopes while cementing were previously reported and do not contradict our interpretation^33^. As the origin of the low-frequency response is unclear, we suggest measuring in future studies, e.g., the electric conductivity of the cementing outflow, and comparing changes to the occurrence of low-frequency responses as a solution to answer this question. Evaluating the displacement efficiency in real-time by tracking fluid interfaces with the help of fiber sensors enables onsite process control. It might significantly reduce the risk of failures in the construction phase.

For the setting of the cement, we demonstrated that the critical hydration phases are determinable despite a strongly heated-up near-field of the well, unstable initial temperature conditions, and the massive cool-down of the borehole due to the cold cement slurry. DTS allows better detecting differences in the individual hydration stage during the cement setting. However, we found evidence that DDSS can solely represent the end of the critical deceleration phase (Fig. 8c). Therefore, a single DDSS measurement could control displacement efficiency and determine the final set and the Wait on Cement. This information is important for all depths, as the cement must withstand the impact of the drill strings along the casing. Due to subsurface ambiguities, a direct WOC statement can only be made by comparing the downhole data with the reference sample and a hydration model.

A combined interpretation of field data and modeled data proved a significant improvement in displacement efficiency^45,46^. Validating the fluid displacement model with real-time downhole data could result in a similar improvement. Despite vast amounts of data, approaches to evaluating real-time fiber sensor data exist^47,48^. We suggest including the fiber optic results and interpretation in the well dossier. As the results of future well-logging campaigns can be ambiguous^17,18^, a comparison with the displacement and hydration history can support the decision for workovers. While we can indicate the exact depth, we must also mention that a single vertical fiber provides no azimuthal information. However, our approaches to improve well integrity are entirely free of additional deployment costs if the borehole is already planned with a permanent fiber optic sensing cable for reservoir monitoring.

## Methods

### Experimental setup

The cement job analysed within this study was part of constructing an injection well at the geothermal site in Munich, Germany. This site is located in the North Alpine Foreland Basin (Bavarian Molasse basin) and explores the hydrothermal reservoir in the Lower Cretaceous (Purbeck layer) and the limestone and dolomite of the Upper Jurassic to a depth of approximately 2800 m TVD.

The fiber optic sensing cable was installed in 2019 by the Geothermie-Allianz Bayern when a 20" and 874 m deep surface casing was deployed for a new injection well^49^. The fiber optic cable is attached to the outside of the casing with special clamps running together with the casing. The cable is a flatpack configuration (13.7 × 30.5 mm) with 2 single-mode fibers (for DDSS) and 2 multi-mode fibers (for DTS)^49^. Mini bends loop the fibers back to the surface at a depth of 699 m. After installation, an OTDR measurement proved the fibers’ integrity and the deployment’s success.

The cement job was conducted as an inner string stinger cementation, pumping fluids through a drill pipe, exiting a float shoe at the bottom of the casing, and rising in the annulus between the casing and formation towards the surface. Preparation began at night with the conditioning of the drilling mud. In the morning, a high-viscosity pill (HV-pill) was pumped to help clean the borehole from drilling mud. A freshwater spacer followed this pill. Finally, the cement was pumped. Shortly after noon, the cement reached the surface with the desired density, and the cement placement was considered done. A UCA test was performed in advance for a bottom hole static temperature of 48°C, and the caliper log was run the day before for volume calculation.

We monitored the cement placement and the 24 h of early hydration with DTS and DDSS measurements. The DTS measurement included roughly 12 h of the borehole’s initial temperature conditions, with a relatively low temporal resolution, to increase the accuracy. The DDSS measurement started with the cement placement. Table 1 shows an overview of the fiber optic measuring units and their key parameters.

**Table 1:** Used fiber optic units and their key parameters.

	DTS	DDSS
Type	AP sensing	hDAS by Aragon Photonics
Parameter	Temperature	Strain rate
Measurement	Raman OTDR	Chirped-pulse Phase OTDR
Temporal resolution	13 min	1000 Hz
Spatial resolution	1 m	1 m with 10 m gauge length
Configuration	Double ended	Single-ended in a loop

### Fiber optic data processing

We processed the fiber optic data with in-house software solutions based on Python standard libraries and obspy^50^. In the first step, we allocated each fiber sensor to its position along the borehole. A double-ended configuration allows this by knowing the depth of the sensor cable’s turning point and identifying the channels precisely at the surface. We then interpolated all other measured channels between these fixed points.

*DTS* We used a cold spray to identify the cable entry into the well at the surface. To ensure a correct calibration, the absolute readings were compared with a downhole pressure/temperature gauge and an independent wireline temperature log in a nearby well (15 m wellhead distance)^49^. We compared the initial temperature conditions to the geothermal gradient. We then examined the temperature change between each measurement as a proxy for the evolution of the hydration’s released heat. Understanding the influence of formation temperature and comparing the temperature change with the characteristic heat rate development during hydration^7^ allowed us to estimate the hydration phases for each depth. We used a Savitzky–Golay filter to reduce the DTS units’ measuring noise.

*DDSS* We identified the channels at the surface with a tap test. The dynamic strain rate data underwent analysis in various frequency ranges utilizing a Butterworth-Bandpass filter. We investigated the vibrational energy within the different frequency bands by applying a root mean square with different-sized rolling windows (n) (Eq. 1).

1 $$RMS = \sqrt {\frac{1}{n}\sum\nolimits_{{i = 1}}^{n} {x_{i}^{2} } }$$

These results are referred to as vibrational energy. The strain rate data was further examined in the frequency and frequency-wavenumber domains to understand the origin of the vibrational energy. A blotchy pattern of irregular size and shapes was quantified using the Python library OpenCV^51^. First, we discretized the data with a 2-bit normalization (threshold = mean of all values). We defined a patch with 30 × 30 values (representing 30 m × 30 min) around each measured value. We determined the number of connected values representing one spot and their average size within this patch.

### Fluid displacement model

We developed a fluid displacement model to understand the rise of fluids in the annulus and to predict the expected height of different fluid interfaces over time. Equation 2 shows how we used the geometry of the borehole annulus ($A\left(\delta \right)$, based on a caliper log) for each current depth $\left(\delta \right)$ to determine the cumulative sum of the cement volume (${V}_{annulus}(d)$) needed to fill each depth interval ($\Delta d$). Before the cement begins to rise in the annulus, the volume of the stinger (${V}_{stinger}$, cementing pipe from the surface to the bottom of the casing), and the volume of the rat hole (${V}_{rat hole}$, volume below the casing) must be filled. However, the rat hole volume is unknown and can only be estimated.

2 $${V}_{annulus}(d)= {V}_{stinger}+{V}_{rat hole}+\sum\nolimits_{\delta ={d}_{bottom}}^{d}A\left(\delta \right)\times \Delta d$$

Once we set a pumping start time for each fluid interface (${t}_{0}$), we calculated the total pumped volume based on the pumping rate (${V}_{pumped}(\tau )$) for each time index ($\tau$) (Eq. 3).

3 $${V}_{pumped}\left(t\right)= \sum\nolimits_{\tau ={t}_{0}}^{t}{V}_{pumped}(\tau )$$

A comparison of both cumulated volumes predicts the height of the fluid interface for each time ($t$). Considering the cross-sectional area of the annulus and the pumping rate, we can calculate the rising velocity for each depth. With a constant pumping rate (${Q}_{pumped}$), we hypothesize that the rising velocity (${v}_{rise}$) can only change with a changing cross-sectional area of the annulus (${A}_{annulus}$) (Eq. 4).

4 $${v}_{rise}= \frac{{Q}_{pumped}}{{A}_{annulus}}$$

We calculate the rise of fluids for the maximum annular volume (accounting for all borehole washouts) and the minimum volume (the bit diameter in the absence of washouts).

### Wait on cement estimation

We tested the same cement composition used cementing to estimate the minimum Wait on Cement. The cementing service conducted a UCA test. Further, the evolution of the S-wave velocity was determined based on a double cross-correlation approach^52^. With the result of the UCA test and the onset of the shear wave velocity, we assume an estimated time in which the setting and the WOC should be reached at the latest. However, the cement was mixed continuously for 4.5 h, so the start of mixing and hydration changes for each depth. We used the result of our fluid displacement model to determine the duration of one pumping cycle and correlated the end of this cycle with the end of the pumping process. Cement close to the surface was mixed when this last cycle began, and cement at total depth was mixed close to the end of the cycle.

## Data Availability

The temperature and vibrational energy data described and analyzed in this article are published via GFZ Data Services (10.5880/GFZ.2.2.2024.001) ^53^. The data will be published via GFZ Data Services when this article is accepted. Raw data and operational data of the cementing service are available from the corresponding author upon reasonable request.
